# Examination of a Low-density Lipoprotein Receptor Relative with 11 Ligand-binding Repeats (LR11) as a Biomarker in Esophageal Cancer

**DOI:** 10.14789/jmj.JMJ22-0008-OA

**Published:** 2022-10-04

**Authors:** TAKAYUKI UCHIDA, MOTOMI NASU, TAKASHI HASHIMOTO, MASAHIKO TSURUMARU, YOSHIAKI KAJIYAMA

**Affiliations:** 1 Department of Esophageal & Gastroenterological Surgery, Juntendo University Graduate School of Medicine, Tokyo, Japan; 1 Department of Esophageal & Gastroenterological Surgery, Juntendo University Graduate School of Medicine, Tokyo, Japan

**Keywords:** esophageal cancer, lipoprotein receptor, LR11, biomarker

## Abstract

**Objectives:**

Some previous studies reported that the levels of a low-density lipoprotein receptor relative with 11 ligand-binding repeats (LR11) was a prognostic marker in some malignant tumors; however, whether LR11 is related to survival in patients with esophageal cancer remains unclear.

**Methods:**

In this study, we measured LR11 in the preoperative serum of 46 patients of esophageal cancer who undergoing surgery using a sandwich enzyme-linked immunosorbent assay (ELISA) method with anti-LR11 monoclonal antibodies. We investigated the correlation between the level of LR11 and survival of patients with esophageal cancer. Clinicopathological data were retrospectively retrieved from our institution's database.

**Results:**

The patients were divided into two groups (low LR11 and high LR11) based on the level of LR11. There was no statistical difference in clinicopathological factors between these two groups. The low LR11 group had a significantly longer overall survival than the high LR11 group.

**Conclusions:**

LR11 can be measured with a relatively simple ELISA and is potentially a new prognostic marker for esophageal cancer.

## Introduction

Eleven ligand-binding repeats (LR11) is a receptor that shares a gene structure with the low-density lipoprotein (LDL) receptor and serves as an essential lipoprotein receptor that regulates the homeostasis of cholesterol metabolism. LDL receptors are thought to be involved in arteriosclerosis as well as in the migration of vascular smooth muscle cells^1)^. It has been reported that the expression of the lipoprotein receptor related protein-1 or the LDL receptor-related protein 1B is an expression of a function-deficient gene family^2, 3)^. This gene family was identified from lung cancer and may be involved in the infiltration of tumor cells or metastasis^2, 3)^. LR11, which is part of the LDL receptor family, has been reported as a marker for Alzheimer's disease and arteriosclerosis^4)^, as well as for leukemia^5)^, and it has been reported that it may also be expressed in the serum of patients with cancer. However, there is no study for association between LR11 and esophageal cancer.

In this study, we examined the relationship between preoperatively collected serum LR11 levels in patients with thoracic esophageal cancer and survival to clarify whether LR11 is a useful prognostic factor for esophageal cancer.

## Methods

This study included a total of 46 patients diagnosed with esophageal cancer who underwent radical esophagectomy with three-field lymphadenectomy via a right thoracotomy and laparotomy at Juntendo University Hospital between September 2005 to December 2006^6)^. Written informed consent was obtained from all enrolled patients. This study was conducted within the guidelines set by the Declaration of Helsinki and approved by the ethical committee of Juntendo Hospital (No. E21-0253). Blood was collected just before surgery. A total of 10 mL of collected blood was centrifuged, and the obtained serum was stored at -80℃.

The serum LR11 levels were measured by the sandwich enzyme-linked immunosorbent assay (ELISA) described below using anti-LR11 monoclonal antibodies. A total of 100 μL of eight-fold diluted serum was dispensed into three wells of an antibody-binding plate (anti-LR11 mouse monoclonal antibody, Sekisui Medical Co., Ltd., Tokyo) that was then left at room temperature (18-26℃) for 2 h. The liquid in the well was discarded, and the well was washed with 350 μL of cleaning solution (buffer solution, Sekisui Medical Co., Ltd., Tokyo). The washing solution was removed, and 100 μL of biotin-labeled antibody solution was added and left at room temperature for 1 h. The solution in the wells was then removed, and then the wells were washed. After washing, 100 μL of tetramethylbenzidine solution (Sekisui Medical Co., Ltd., Tokyo) was added and left to stand at room temperature, away from light to luminesce. Next, 100 μL of stop solution (sulfuric acid, Sekisui Medical Co., Ltd., Tokyo) was added to stop the reaction. A microplate reader was used to measure the absorbance at a wavelength of 450 nm.

The serum LR11 concentration measured by same method was reported around 10 ng/mL in most healthy individuals^7, 8)^; thus, we divided the patients into a low LR11 group (LR11 levels below 10 ng/mL) and a high LR11 group (LR11 levels of 10 ng/mL or higher) and compared the two groups.

We examined the relationship between LR11 levels and clinicopathological findings of esophageal cancer. Additionally, we also examined its relationship with serum total protein (TP) level and albumin (Alb) level, which reflect nutritional status, and serum total triglyceride (TG) level and total cholesterol (T-Cho) level in serum. Treatment strategies were decided according to the Union for International Cancer Control (UICC) TNM classification 7^th^ edition for esophageal cancer^9)^ in 2005 to 2006, so we analyzed based on this staging. Japanese Classification of Esophageal Cancer 11^th^ edition was used for tumor location and vascular invasion evaluation^10, 11)^. For statistical analyses, we used Kruskal- Wallis test and the Kaplan-Meier estimate for survival analysis (IBM SPSS Statistics ver.26).

## Results

### Patients' characteristics

**Figure 1:**
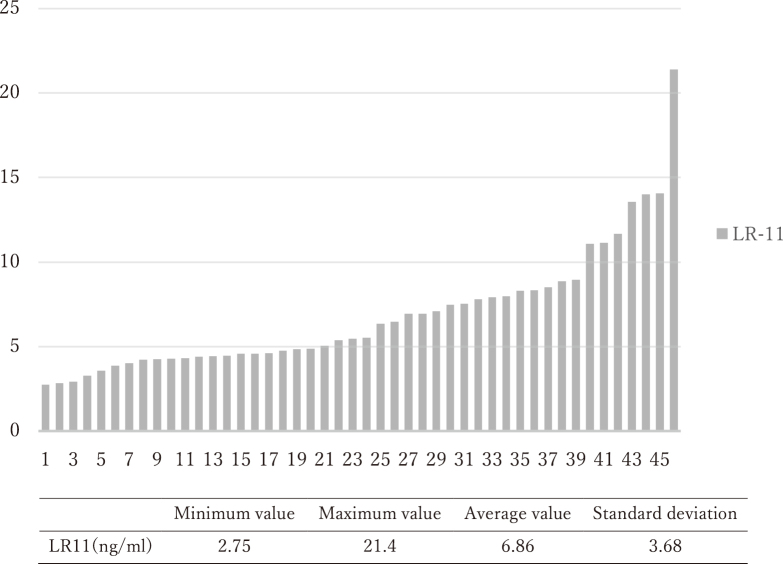
Distribution of serum LR11 levels in patients with esophageal cancer

Patients' characteristics are shown in Table 1. The serum LR11 levels ranged from 2.75 ng/mL to 21.4 ng/ mL. The average value across all patients was 6.86 ng/mL, and the standard deviation was 3.68 ng/mL (Figure 1).

**Table 1:** Clinicopathological background

		Low-level group (n)	High-level group (n)	
Gender	Male	34	5	NS
	Female	5	2	
Main tumor location*	Ce	0	1	NS
	Ut	7	0	
	Mt	19	3	
	Lt	11	3	
	Ae	2	0	
Histologic type				NS
Squamous cell carcinoma				
	Well	15	2	
	Mod	18	2	
	Poor	4	2	
Carcinosarcoma		1	1	
Malignant melanoma		1	0	
Depth of tumor invasion**	pT1	14	0	NS
	pT2	7	1	
	pT3	14	4	
	pT4	4	2	
Lymph node metastasis**	N0	12	3	NS
	N1	12	2	
	N2	11	0	
	N3	4	2	
pStage**	Ⅰ	8	0	NS
	Ⅱ	11	4	
	Ⅲ	16	2	
	Ⅳ	4	1	

Ce: Cervical esophagus, Ut: Upper thoracic esophagus, Mt: Middle thoracic esophagus, Lt: Lower thoracic esophagus, Ae: Abdominal esophagus, Well: Well differentiated squamous cell carcinoma, Mod: moderately differentiated squamous cell carcinoma, Poor: poorly differentiated squamous cell carcinoma *: Japan Esophageal, S; Japanese Classification of Esophageal Cancer, 11^th^ Edition **: UICC TNM Classification of Malignant Tumours, 7^th^ ed NS: Not Significant

After dividing the patients into the low-and high-LR11 level groups (cutoff value of LR11: 10 ng/mL), there were 39 patients in the low LR11 group and seven in the high LR11 group. There were no significant differences between the two groups in patients' characteristics.

### Relationship between serum LR11 levels and pT staging

**Figure 2:**
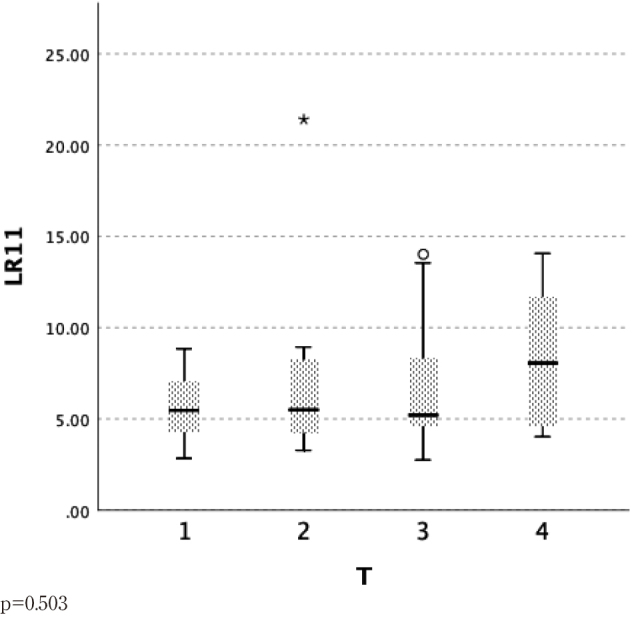
Depth of tumor invasion (T factor)* and serum LR11 levels in patients with esophageal cancer T: UICC TNM Classification of Malignant Tumors, 7^th^ edition

We examined the relationship between pT staging (classified as pT1, pT2, pT3, and pT4) and serum LR11 levels (Table 1). The serum LR11 levels tended to be lower in patients staged pT1, and to rise with increased tumor invasion degree; however, no significant differences were found (Figure 2).

### Relationship between serum LR11 levels and pN staging

**Figure 3:**
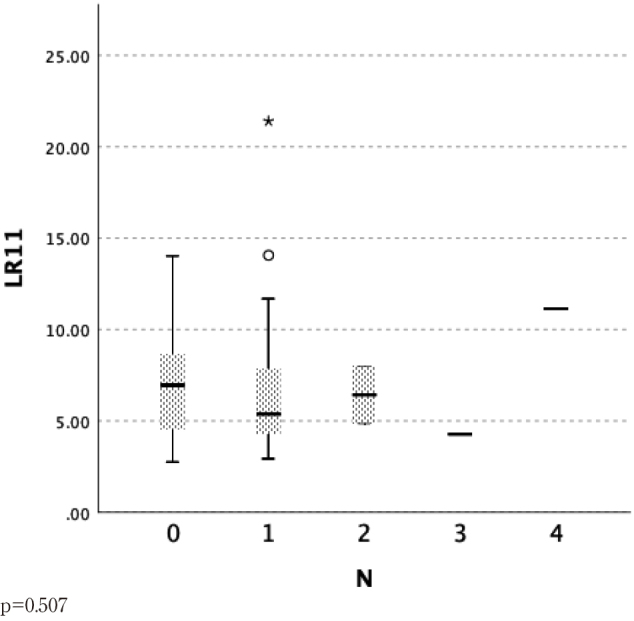
Lymph node metastasis (N factor)* and serum LR11 levels in patients with esophageal cancer *: UICC TNM Classification of Malignant Tumors, 7^th^ edition

Regarding pN, the mean ± standard deviation values of serum LR11 in each group characterized as pN0, pN1, pN2 and pN3 showed no significant differences (Figure 3).

### Relationship between serum LR11 levels and vascular invasion

**Figure 4:**
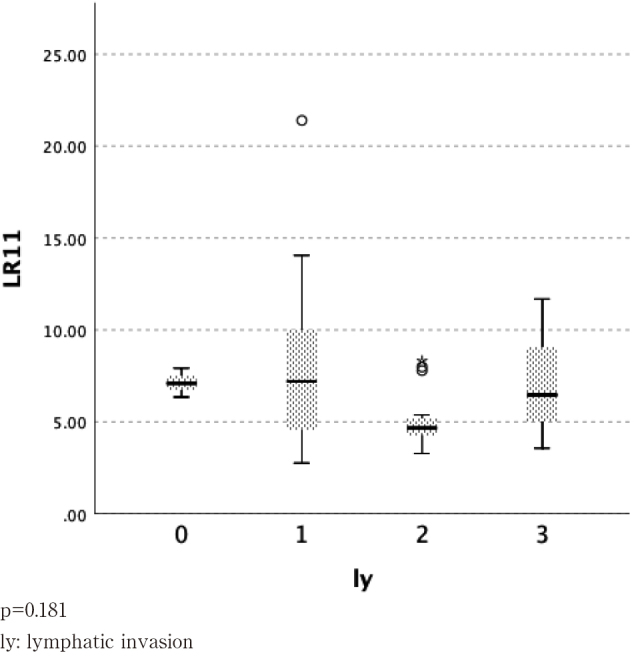
Lymphatic invasion (ly)*^2^ and serum LR11 levels in patients with esophageal cancer *^2^: Japanese Classification of Esophageal Cancer 11^th^ ed

**Figure 5:**
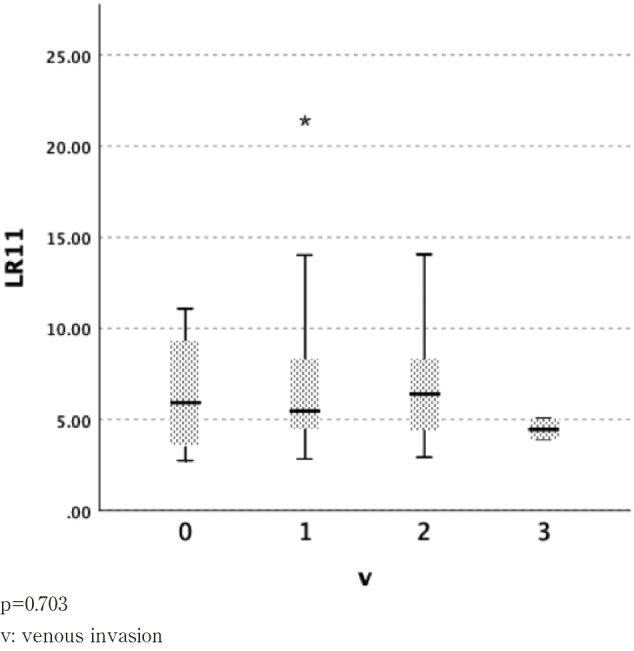
Venous invasion(v)*^2^ and serum LR11 levels in patients with esophageal cancer *^2^: Japanese Classification of Esophageal Cancer 11^th^ ed

We examined serum LR11 by classifying patients according to the degree of lymphatic invasion into ly0, ly1, ly2, and ly3 (Figure 4) and according to degree of venous invasion into v0, v1, v2, and v3 (Figure 5). There were no significant differences between these groups.

### Relationship between serum LR11 levels and blood biochemistry

**Figure 6:**
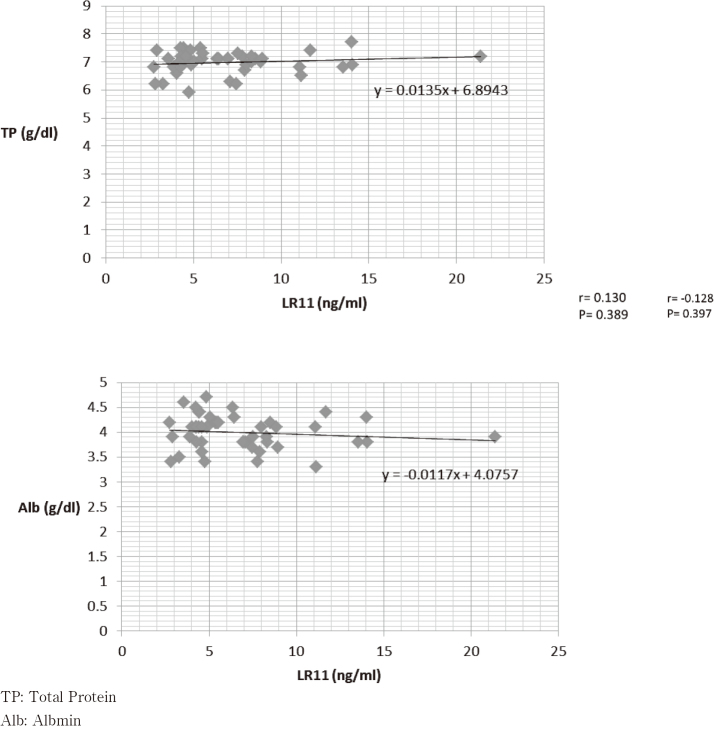
Total protein/albumin and LR11

**Figure 7:**
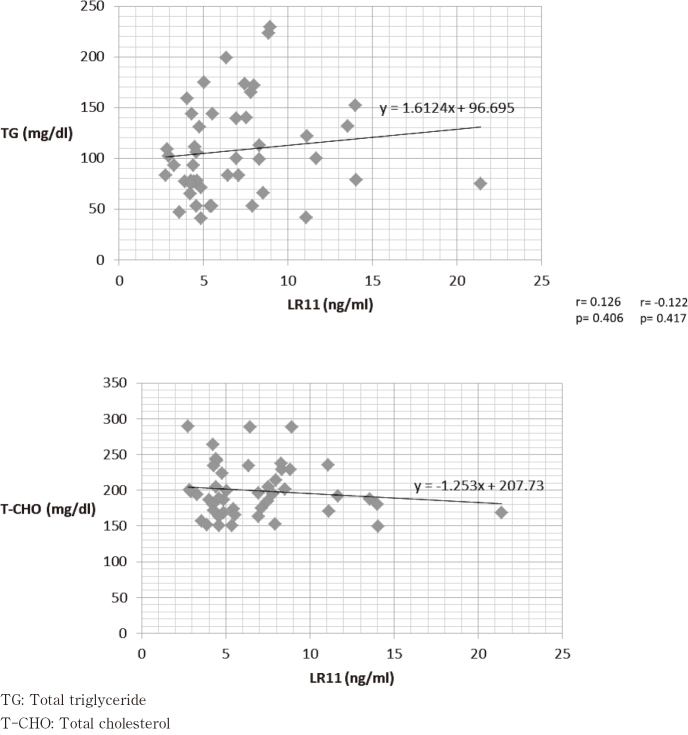
Total triglyceride / Total cholesterol and LR11

We examined the correlation between each of the blood biochemistry findings, as TP, Alb, TG, and T-Cho with serum LR11 levels; however, the results showed no significant differences for each value (Figure 6, 7).

### Relationship between serum LR11 levels and survival

**Figure 8:**
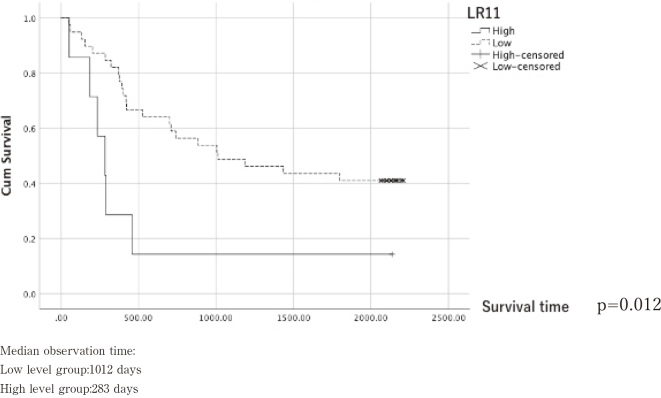
Overall Survival

We compared overall survival of 39 patients in the low LR11 group and seven patients in the high LR11 group using the Kaplan-Meier estimate. Results showed that the 5-year survival rate was 41% in the low LR11 group and 14.3% in the high LR11 group, with a significantly worse prognosis for the high LR11 group (p=0.012, Figure 8).

## Discussion

LR11 was discovered by Jiang et al. in 2008 as an important factor involved in the differentiation of undifferentiated vascular smooth muscle cells^12)^. In 2010, Takahashi et al. found that serum soluble LR11（sLR11） was an indicator of coronary artery stenosis^13)^. Furthermore, it has been noted in recent years that sLR11 has characteristics as a biomarker for malignant tumors, where its levels increased with the exacerbation of acute leukemia, normalized with remission, and increased significantly with tumor infiltration into the bone marrow^14, 15)^. Not only in the serum, LR-11 levels reported significantly higher in the bile from biliary tract cancer and pancreas cancer patients than in those with benign diseases^16)^.

In this study, we hypothesized that serum LR11 level should rise in esophageal cancer patients, and examined the expression of LR11 in the serum of patients to evaluated the clinical significance of LR11. Thereby, the high serum LR11 level group had a significantly worse prognosis compared to the low LR11 group.

Firstly, the serum levels of patients with esophageal cancer were 6.86 ± 3.68 ng/mL; this value was relatively low when compared to values for patients with non-Hodgkin's lymphoma (17.7 ± 22.6 ng/mL), acute lymphocytic leukemia (73.5 ± 93.5 ng/mL), and acute myeloid leukemia (26.8 ± 29.1 ng/mL), where LR11 is already used as a biomarker^15-25)^. It can be said that the value of serum LR11 in patients with esophageal cancer was lower than people without disease because the mean value of serum LR11 was reported to be around 10 as described above. We speculated that it was from patients' malnutrition because serum LR11 was related to metabolism of cholesterol and patients with esophageal cancer often become undernourished caused from esophageal stenosis. We compared LR11 levels with TP/ Alb, which reflects the nutritional status of the patient, and TG/ T-Cho, which reflects lipid metabolism in the patient; however, the results showed no significant differences. Therefore, we suspect that relatively low level of LR11 might be from differences between gastrointestinal solid cancers and hematological malignancies^26)^. Recently, in patients with bile tract cancer and pancreatic cancer, bile sLR11 suggested to release from the cancer cell, and may reflect the characteristics of the microenvironment such as hypoxic conditions, and rapid cell proliferation^14-16)^. Similarly, esophageal cancer might be affected by their microenvironment, so that it needs further study.

Based on our data, the level of serum LR11 are likely to be related to pT status, but not associated with pN, lymphovascular invasion, and other nutritional or lipid markers in blood. It is interesting that only pT status is likely to be related to serum LR11 because most prognostic biomarkers tend to be related to other significant prognostic markers like pT, pN, and lymphovascular invasions. We speculate that serum LR11 might not be related to the malignant potential but might be related to other factors such as size of tumors which could be influenced by food intake. In this study, we were not able to get information about how much weight patients lost before esophagectomy.

This study has some limitations. First, this study utilized a retrospective design. Second, the sample size was very small, and short period of observation. Moreover, the effect of another clinical features like adjuvant therapy, hyperlipidemia, and atherosclerosis need to be validated. Further, pathological evaluation of LR-11 expression in tumor tissue remains to be clarified, too.

In Conclusion, LR11 could be measured using the relatively simple ELISA method and is expected to be used as a new prognostic predictor for esophageal cancer.

## Funding

The author(s) received no financial support for the research.

## Author contributions

TU corrected blood samples, interpreted the patient data, and was a major contributor in writing the manuscript. The article was revised by MN and all authors read and approved the final manuscript.

## Conflicts of interest statement

The authors declare that they have no conflict of interest.
